# Case Report: Silicatosis in a Carpet Installer

**DOI:** 10.1289/ehp.9691

**Published:** 2007-03-02

**Authors:** Jaime Szeinuk, Elizabeth J. Wilk-Rivard

**Affiliations:** Mount Sinai–Irving J. Selikoff Center for Occupational and Environmental Medicine, Department of Community and Preventive Medicine, Mount Sinai School of Medicine, New York, New York, USA

**Keywords:** carpet installers, mycobacteria, silicatosis, silicosis, talc, tuberculosis

## Abstract

**Context:**

Chronic exposure to talc in the course of carpet installation can result in pneumoconiosis.

**Case presentation:**

We present a case of a young carpet installer who was diagnosed with silicatosis of the lung. Review of occupational history revealed that the patient had been working as a carpet installer for approximately 15 years, since he was 15 years of age. The patient was exposed to talc in the course of his work.

**Discussion:**

Exposure to talc in the course of carpet installation has not been reported as a possible cause of pneumoconiosis. In this article we review different causes of silicatosis and discuss chronic exposure in the course of carpet installation and development of pneumoconiosis. In addition, we also review the relevance of mycobacterial infection in cases of silicosis and silicatosis.

**Relevance to Clinical or Professional Practice:**

Exposure to talc in the course of carpet installation should be added to conditions that can cause pneumoconioses, specifically silicatosis of the lung.

## Case Presentation

### Clinical history

The patient, a 31-year-old male, was evaluated at the Mount Sinai–Irving J. Selikoff Center for Occupational and Environmental Medicine on 22 March 2005. He complained of shortness of breath at rest and with exertion, dry cough, and cough with phlegm. Additional symptoms included fatigue, chest tightness, wheezing, and decreased ability to perform physical activity. The patient had begun experiencing breathing difficulty about 2 years before his initial visit, and his symptoms had progressed over time. In 2004 he became more symptomatic and was examined by a nurse practitioner. He was diagnosed with bronchitis and prescribed an albuterol inhaler, salmeterol/fluticasone diskus, and oral prednisone for several days. At the time, it was recommended that he obtain a chest X ray, but he did not follow up on this recommendation. As a result of the above-mentioned treatment, his symptoms partially improved and he continued to work, albeit only part time because of continuing respiratory symptoms. In the beginning of 2005, the patient was examined by his primary care physician, who sent him for a chest X ray and referred him to a pulmonologist. The pulmonologist then ordered a chest computed tomography (CT) scan, bronchoscopy, and lung biopsy and referred the patient to our clinic for evaluation. At the time of his visit to our clinic, the patient was not taking any medication.

Review of systems was unremarkable. His past medical history revealed orchidopexy at 9 years of age. He denied having allergies to any medications or to any other exposure. At the time of his visit he was actively smoking 20 cigarettes/day on average; he began smoking at age 11. He described himself as a moderate drinker.

### Occupational history

The patient had been a carpet and floor installer for 15 years. He began working at the age of 15 and he had worked for different carpeting and flooring companies, mostly small family businesses.

The patient’s major occupation was the installation of new carpeting and the removal of old carpets, stripping and patching floors using cement and water, and scraping and cleaning floors. In the process of his work, he used adhesives and glues on floors. The patient mentioned that there was usually a substantial amount of talc present when installing new carpeting. He was not familiar with any material safety data sheets relevant to his job. The patient had not received proper job training, and he did not use any respiratory protection or other personal protective equipment when doing his work.

### Physical examination

On physical examination, the patient’s blood pressure was 120/75 mmHg, his pulse was 90 beats/min, and his respiratory rate was 28/min, with a fast, regular but shallow breathing pattern. His height was 6 feet and his weight was 158 lb, for a body mass index of 21.1 kg/m^2^. Examination of his head and neck was normal. His lungs were clear to percussion and auscultation, and examination of the heart showed regular and rhythmic heart sounds and no murmurs. His abdomen was soft to palpation, with no tenderness. Liver span was normal, and no megalies or masses were noted. An examination of the patient’s lower extremities revealed normal peripheral pulses and no edema. Musculoskeletal examination was normal; neurological examination was nonfocal, and his skin was clear.

### Laboratory and X-ray evaluation

**Figure 1:**
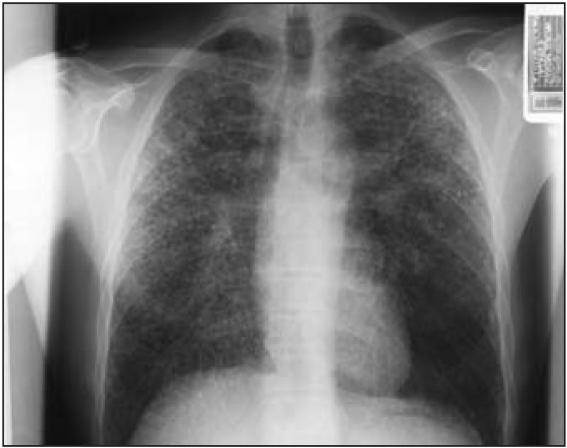
Postero-anterior chest X ray demonstrating bilateral nodular regular-nodular infiltrates.

**Figure 2:**
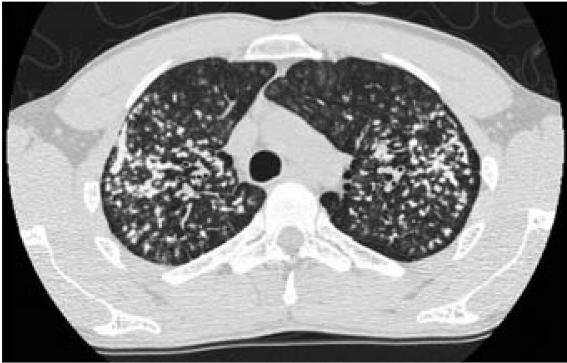
Chest CT scan section at the apices of the lungs indicating coalescence and rounded opacities.

**Figure 3:**
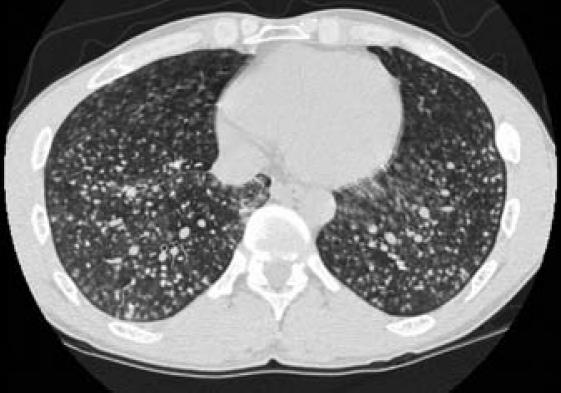
Chest CT scan section of the mid-chest CT showing diffuse nodular infiltrates.

Chest X rays on 16 February 2005 showed a diffuse, extensive, bilateral regular-nodular infiltrate, with nodules measuring < 5 mm in diameter. Roentgenographic interpretation according to the International Labour Organization (ILO) classification for pneumoconiosis (ILO 2002) revealed rounded regular opacities classified as q/r in shape and size (q, 1.5–3 mm; r, 3–10 mm), located throughout both lungs, with a degree of profusion of 3/3 (on scale of 0/– to 3/+) (Figure 1). No large opacities were noted, and no pleural abnormalities were described; coalescence of small opacities was described. A chest CT scan on 24 February 2005 revealed multiple nodular lesions throughout both lungs, from the apices to the bases (Figures 2 and 3), with coalescence of opacities and formation of conglomerates in both upper lobes. No evidence of consolidation or mediastinal or axillary lymphadenopathy was seen, but it was noted in the record that the hilar nodes could not be evaluated because of the use of a noncontrast technique. No pleural effusion was visualized. Occasional bullous changes were described in the apices of the lungs. Blood tests revealed a normal C-reactive protein. The erythrosedimentation rate was 10 mm/hr.

### Pulmonary function tests

Pre- and post-bronchodilator spirometry was performed on 22 March 2005. Prebronchodilator forced vital capacity (FVC) was measured at 3.09 L (55% of predicted), and prebronchodilator first-second forced expiratory volume (FEV_1_) was 1.97 L (43% of predicted); the FEV_1_/FVC ratio was 64% (predicted 82%). Postbronchodilator, the patient’s FVC improved by 460 mL (15%), and his FEV_1_ improved by 1.02 L (26%). This study was interpreted as a mixed obstructive and restrictive impairment with significant improvement after inhaled bronchodilator.

### Pathology reports

The patient underwent bronchoscopy with transbronchial biopsy on 7 March 2005. Bronchoalveolar lavage was negative for malignant cells, but the culture of lavage was positive for *Klebsiella oxytoca* and *Mycobacterium avium-intracellulare*. The transbronchial biopsy was referred for consultation. The pathologist reported

… a dense interstitial histiocytic infiltrate with numerous multinucleated, foreign-body type giant cells. Numerous sheet-like refractile and strongly polarizable particles are present within the histiocytes. The appearance fits with a form of silicate pneumoconiosis, most likely of the type due to talc, although mica and kaolin can cause a similar appearance.

No further pathology reports or results of any special pathology study (such as electron microscopy or spectroscopy studies) were available.

### Case management

The nature and the implications of the diagnosis were explained to the patient. The patient was urged to stop smoking and advised to change his job and avoid exposure to respiratory irritants. The patient was referred for full pulmonary function tests with plethysmography and diffusing capacity. He received a prescription for an albuterol inhaler, and his follow-up appointment was scheduled at our clinic for 3 weeks after the initial evaluation. It was further recommended that the patient continue to follow-up with his pulmonologist and primary care physician. Despite multiple phone calls and written letters, the patient did not attend any appointment, neither at our clinic nor with the pulmonologist or his primary care physician. The impression of the treating physicians was that he had understood the nature of his disease but was unable to follow medical recommendations.

## Discussion

Based on medical and occupational history, physical examination, and results of laboratory and pathology tests, this patient was diagnosed with silicatosis, most probably talcosis. The cause of his lung disease was thought to be related to his working as a carpet installer.

Silicatosis is a type of pneumoconiosis caused by a variety of silicates from diverse environmental and occupational sources (Kales and Mark 1995). Although silicatosis is related to silicosis both clinically and pathologically, it is usually milder than silicosis and less fibrogenic (Silicosis and Silicate Disease Committee 1988). Silica is a mineral composed of silicon and oxygen; silicates, in turn, originate when silicon combines with other anions and oxygen (Morgan 1995). Inherent properties of silicates make them useful as fire retardants, fillers, cation exchangers, catalysts, and construction materials for use as building stone, road aggregate, and light-weight aggregate for concrete (Short and Petsonk 1996). Examples of silicates include bentonite, mica (potassium aluminum silicate), feldspar (aluminum silicate with potassium and sodium), talc (magnesium silicate), and kaolin (hydrated aluminum silicate). Talc, which was recognized by the pathologist who interpreted this patient’s biopsies as the most probable cause for the patient’s pneumoconiosis, is not a uniform material (Short and Petsonk 1996).

Talc can be tabular, granular, fibrous, or platy, but it is usually crystalline, flexible, and soft. Commercial talc in the United States comes from > 10 states, with New York, California, Texas, and Vermont being the major producers (Morgan 1995; Short and Petsonk 1996). Commercial talc is usually contaminated with other minerals. Talc has a wide variety of uses: paint, paper, ceramics, cosmetics, roofing products, textile material, rubber, lubricants, corrosion proofing, fire-extinguishing powders, water filtration, insecticides, dusting powders, spackling and patching compounds, and asphalt products. More than 500 different products are sold under the name of “talc” (Morgan 1995; Rom 1998). Contribution from talc exposure to increased mortality due to nonmalignant respiratory disease has been documented in several studies of talc workers (Coggiola et al. 2003; Honda et al. 2002; Wild et al. 2002). These studies confirm an association between cumulative exposure to talc and respiratory disease. However, even a relatively short, intense exposure to talc can result in diffuse pulmonary disease, with a latency period of more than 40 years (Gysbrechts et al. 1998). According to Feigin (1986) and Rom (1998), talc-induced pulmonary disease has four distinct manifestations, the first of which is talco-silicosis, which is similar to silicosis. Second, talcoasbestosis, which closely resembles asbestosis, is produced by crystalline talc contaminated with asbestos fibers. Third, talcosis, caused by inhalation of pure talc, may include acute or chronic bronchitis as well as interstitial inflammation; radiographically it appears as reticular or nodular abnormalities, and functionally it causes small airway obstruction. The fourth form is caused by intravenous injection of talc. According to Morgan (1995), pure talc leads to a mixture of rounded and irregular opacities that appear in the middle zones of the lungs and are often perihilar in distribution. The opacities slowly spread both up and down the lung fields. Large opacities that result from coalescence of the small opacities may be present. In CT scans of talc pneumoconiosis, the predominant abnormalities have been described as small centrilobular and subpleural nodules and conglomerated masses containing focal areas of high attenuation (Chong et al. 2006; Marchiori et al. 2004). Pulmonary function studies of patients with talcosis reveal restrictive, obstructive, or mixed patterns (Avolio et al. 1989).

Kaolin, also known as China clay, is a soft white material that is used in the manufacture of paper products, refractory materials, and ceramics and as a filler in plastics, rubber, and paints (Short and Petsonk 1996). In kaolin workers, chest radiographic manifestations are those of rounded and irregular opacities, and pulmonary function studies show reductions in FVC, FEV_1_, and peak flow rates. The potential for kaolin dusts to induce lung damage in the absence of crystalline silica contamination is not universally accepted (Short and Petsonk 1996). Some researchers, however, believe that pure kaolin, in the absence of silica, can induce pneumoconiosis, usually nodular in appearance, with the possibility of coalescence and formation of large opacities (Morgan 1995). Kaolin is far less fibrogenic than silica and is not associated with significant ventilatory impairment (Morgan 1995). Mica has electrical and thermal properties that make it useful as liner for steam boilers, in optical instruments, in oil-well drilling, as artificial snow and flocking for Christmas ornaments, in roofing material, as a filler for asphalt and plaster, in ceiling tile and wallboard joint cements, and in electrical insulation (Short and Petsonk 1996).

Mica dust can cause pneumoconiosis (Short and Petsonk 1996; Zinman et al. 2002), characterized by nodular and reticular infiltrates especially in the lower lung fields. However, pneumoconiosis due to mica appears to be rare (Morgan 1995).

Our patient’s trade was carpet and floor layering. Carpet installation requires basic carpentry skills and physical strength. Installing carpeting and juxtaposition of seams requires the use of adhesives, which are applied with a trowel to the underlying floor. These adhesives may contain acrylic resins (acrylates and methacrylates), styrene, butadiene, rubber latex, and halogenated hydrocarbons. Exposure to products used in carpet layering has been associated with skin, eye, upper and lower respiratory, and central nervous system effects, as well as with musculoskeletal disease (Medora 1997). Our patient, in relation to his clinical diagnosis, mentioned that there was a substantial amount of talc present when installing new carpets. Talc is cited in some commercial sites as a carpet backing and as filler when preparing subfloors for carpet installation (Eager Plastics Inc. 2006). Exposure to methacrylates can result in hypersensitivity pneumonitis, as has been reported especially in dental technicians (Scherpereel et al. 2004). Although hypersensitivity pneumonitis may cause radiographic abnormalities, usually the shape, size, and degree of these would not be as extensive as the ones seen in our patient. In addition, pathology studies would not show polarizable particles in such cases.

Cultures from bronchial lavage of our patient were positive for *M. avium-intracellulare* and *K. oxytoca*. Both typical tuberculosis and atypical mycobacteriosis have been described as a complication of pneumoconiosis. The association between silicosis and tuberculosis, for example, has been described since the times of Agricola in the sixteenth century (Snider 1978). The incidence of mycobacterial infection among patients exposed to high concentration of silica has been estimated at 25% (Fujita et al. 2004). Studies have confirmed that tuberculosis is a major cause of morbidity and death among silicotics; the risk of tuberculosis infection and disease is higher in silicotics than in the general population; the incidence of active tuberculosis in chronic silicosis increases in direct proportion with the increase in profusion of silicotic nodules; and the presence of tuberculosis accelerates lung damage caused by silica particles [American Thoracic Society (ATS) 1997a; Snider 1978; Ziskind et al. 1976]. This increased susceptibility to tuberculosis among silica patients has been explained by silica-induced reduction in cell-mediated immunity with alteration in lymphocyte subsets and serum immunoglobulin levels (Rimal et al. 2005) or by impaired function of lung macrophages due to silica (Lowrie 1982).

Nontuberculous mycobacteria disease accounts for a considerable proportion of the mycobacterial disease seen in silicotic patients, especially in the industrialized world, and has also been linked to worsening the clinical course of silicosis (ATS 1997a; Corbett et al. 1999; Fujita et al. 2004; Ziskind et al. 1976). Nontuberculous mycobacterial infection has also been reported in talcosis (De Coster et al. 1996). The ATS has published diagnostic criteria for disease caused by nontuberculous mycobacteria (ATS 1997b). Radiographic features that strongly suggest tuberculosis in patients with silicosis include a rapid progression of X-ray abnormalities, especially in the upper lung fields; the appearance of a cavity; the development of bronchial stenosis or occlusion or of pleural or pericardial effusion; and the presence of any massive unilateral, nonretractile opacity (Fujita et al. 2004; Snider 1978). Most patients with mycobacterial-disease–complicating pneumoconiosis present with clinical signs and symptoms such as fever, night sweats, malaise, and worsening respiratory findings (Snider 1978), although the disease could be paucisymptomatic in many patients (Solomon 2001). A recent review of nontuberculous mycobacteria isolation in respiratory samples of non-AIDS adult patients showed that only 16% of those patients in whom a positive sample for nontuberculous mycobacteria had been identified were ultimately diagnosed with pulmonary disease (Marinho et al. 2005).

In the patient in the present study, we felt that the absence of clincal signs, of worsening pulmonary radiographic infiltrates, and of frank granuloma formation in the pulmonary biopsy strongly argued against active myco-bacterial infection being the cause or contributing to the nodules observed in the films. In addition, with the available information, we determined that this patient did not fully fulfill the criteria for diagnosing nontuberculous mycobacterial lung infection, as recommended by the ATS. However, we cannot rule out a role of mycobacterial infection in causing the degree of profusion of abnormalities shown in this case. The patient’s pulmonologist also agreed with this conclusion, and stated that the patient “may need treatment” in the future.

Cultures of bronchial lavage in this patient also showed *K. oxytoca,* an opportunistic pathogen found in the environment and in mammalian mucosal surfaces. *K. oxytoca* is usually related to antibiotic-associated diarrhea and nosocomial infections (Decre et al. 2004). Given the fact that at no time did the patient present any signs of acute infection, the above findings point to contamination as the most probable cause of this culture result.

Upon reviewing this patient’s occupational history, clinical presentation, and pathology results, it appears that exposure to talc in the course of his occupation as a carpet layer resulted in nodular opacities in the chest CT scan that were further identified as silicate pneumoconiosis on lung biopsy. To our knowledge, this is the first report of such association.

This young patient presented severe pulmonary radiographic and functional findings. In addition to his occupational exposure, he reported a significant smoking history. Most likely, concurrence of smoking from an early age and his occupational exposure contributed to the expression and progress of his disease. Association of smoking and asbestos exposure, for example, has shown that smokers have greater disease magnitude than equally exposed nonsmoking workers (Kilburn et al. 1986; Kilburn and Warshaw 1992; Ohar et al. 2004). In silicosis, obstructive impairment in pulmonary function testing is caused by chronic bronchitis as a result of nonspecific dust effects and smoking (LaDou 2004).

In summary, in this case of a young carpet installer who was diagnosed with silicatosis of the lung, a review of his occupational history revealed that the patient was exposed to talc in the course of his occupation. Carpet installation should be added to the causes of pneumoconioses, specifically silicatosis of the lung.
